# Evaluation of Pain Prevalence in Children Who Experienced Perinatal Hypoxia-Ischemia Events: Characteristics and Associations With Sociodemographic Factors

**DOI:** 10.7759/cureus.46359

**Published:** 2023-10-02

**Authors:** Giovanna Maria G Von Adamovich, João Antonio G Bastos Torres, Felipe S Vianna, Penha C Barradas, Beatriz F Alves de Oliveira, Nivaldo R Villela, Maura Calixto C De Rodrigues, Guilherme C Montes

**Affiliations:** 1 Medical School, Faculty of Medicine, Universidade do Estado do Rio de Janeiro, Rio de Janeiro, BRA; 2 Internal Medicine, Faculty of Medicine, Universidade do Estado do Rio de Janeiro, Rio de Janeiro, BRA; 3 Pharmacology and Psychobiology, Instituto de Biologia Roberto Alcantara Gomes, Universidade do Estado do Rio de Janeiro, Rio de Janeiro, BRA; 4 Epidemiology and Public Health, Fiocruz Regional Office of Piauí, National School of Public Health, Oswaldo Cruz Foundation, Piauí, BRA; 5 Anesthesiology, Pain Medicine, Faculty of Medicine, Universidade do Estado do Rio de Janeiro, Rio de Janeiro, BRA; 6 Pediatrics, Neonatology, Faculty of Medicine, Universidade do Estado do Rio de Janeiro, Rio de Janeiro, BRA; 7 Pharmacology and Psycobiology, Instituto de Biologia Roberto Alcantara Gomes, Universidade do Estado do Rio de Janeiro, Rio de Janeiro, BRA

**Keywords:** asphyxiated, premature, hypoxia-ischemia, children, pain

## Abstract

Introduction: Pain in children who suffer from hypoxia-ischemia (HI) events is still not widely studied. Hypoxia-ischemia is characterized by the momentary or permanent cessation of blood flow and, consequently, of oxygen supply, becoming the main cause of encephalopathy in children. Hyperalgesia was identified in animals undergoing prenatal hypoxia-ischemia by researchers from the Universidade do Estado do Rio de Janeiro (UERJ). Premature and asphyxiated newborns have been admitted to the neonatal intensive care unit (NICU) of Pedro Ernesto University Hospital (HUPE) in Brazil and are monitored by the Outpatient Follow-up of High-Risk Newborns Project (SARAR), but no pain assessment was performed.

Objective: To assess pain in children born in high-risk situations, such as prematurity and perinatal asphyxia, with higher chances of perinatal HI, discharged from the NICU/HUPE, and followed by SARAR.

Methodology: The study was approved by the HUPE Research Ethics Committee. The epidemiological, descriptive, cross-sectional study started in 2021 and finished in 2023, with the application of the pain assessment tool or instrument adapted from the Lübeck Pain-Screening Questionnaire to the caregivers and with the collection of growth and development data. The population consisted of asphyxiated infants born with a gestational age greater than 35 weeks and submitted to the Therapeutic Hypothermia protocol and premature infants discharged from the NICU between two (gestational age 1 (GA1)) and 12 years old. For most of them, pain prevalence was assessed according to its frequency and intensity, as were sociodemographic variables of the child and mother, neural alterations, and the Children's Developmental Scale (DENVER II). The percentage differences between the evaluated factors and the presence of pain were performed using Fisher's exact test and medians using the non-parametric Wilcoxon rank-sum test, both appropriate for the small sample of children. Significance levels of 10% were considered for trends and 5% for statistically significant differences.

Results: Of the 86 children included in our search, 26 (30%) were born with a gestational age greater than 35 weeks and diagnosed with perinatal asphyxia (hereinafter referred to as the asphyxiation group), and 60 (70%) were premature. Pain was reported by 22 (25%) children, of whom 54.4% reported moderate or severe pain. The head and abdomen were the most reported sites (36%). Differences were observed in the percentage distribution of pain between asphyxiates and premature infants (11% vs. 32%; p-value 0.061 on the Fisher test) and between females and males (34% vs. 17%; p-value 0.085 on the Fisher test). Black and Brown children had higher median pain scale values than White children (p-value < 0.027, Wilcoxon rank sum test).

Conclusion: There is a higher prevalence of pain in girls, in the head, in premature infants, and greater intensity among Black and Brown children. Therefore, knowing the pain profile can help improve their quality of life by offering treatments.

## Introduction

Pain is a common problem among children and adolescents, demanding high costs for health services and the population [1,2]. However, it is still not so widely studied in the pediatric population, being limited to analyzing restricted groups of children according to their age and the occurrence or not of the pain, not referring to parameters such as frequency, duration, and intensity of pain [1]. Among the factors that may be related to pain at very early times in life, hypoxic-ischemic events stand out [3]. Hypoxia-ischemia (HI) is characterized by the momentary or permanent cessation of blood flow and, consequently, of oxygen supply, becoming the main cause of encephalopathy in children. In children with a previous history of HI, reduced myelination, astrogliosis, decreased cortical and motor development [4], and increased sensitivity to pain is observed [3]. Pain, particularly chronic pain, can accompany these children's lives due to alterations in pain signaling modulation.

Studies developed in animal models submitted to prenatal HI showed hyperalgesia, alterations in the distribution of the neuronal nitric oxide synthase enzyme (nNOS), and astrogliosis in the periaqueductal gray matter [5]. Lesions observed in children who suffer perinatal HI events are reproduced in animal models, such as reduction of oligodendrocytes [6, 7], astrogliosis, and microgliosis [7,8]. In short, cellular elements involved in neuroinflammation seem to have been altered for a long time [8]. Considering the results in the experimental model pointing to increased hyperalgesia in HI animals, it becomes important, and likely to have an impact on future quality of life, to assess pain in children with a history of HI.

The population of newborns discharged from neonatal intensive care units (NICU), particularly premature and asphyxiated ones, suffer from HI events throughout the entire perinatal period, with varying degrees of severity. Premature infants with very low birth weight and extremely low birth weight (weights below 1,500 g and 1,000 g, respectively) are known to have a greater chance of progressing with changes in their growth and development, from major sequelae such as cerebral palsy, blindness, and deafness to cognitive and behavioral problems and learning, and are considered vulnerable to perinatal HI insults [9]. Of the asphyxia patients who survive HI (about 80% to 85%), 25% develop permanent neurological consequences [9-13], such as concentration deficit, cognitive delay [14-16], motor and perceptual dysfunctions, hyperactivity [10-16], and, in more severe cases, epilepsy and cerebral palsy [11]. Improved neuroprotective care and practices that aimed to reduce the risk and severity of brain injury in this group of vulnerable babies resulted in better survival, but with some form of residual neuro-developmental sequelae, although less severe [17-20]. The treatment of HI is not yet standardized or universally protocoled, but therapeutic hypothermia has been used and intensively investigated, having beneficial effects on harm reduction in newborns with 35 weeks’ gestation or more who suffered hypoxic-ischemic events [21,22].

The main objective of our study was to evaluate the presence of pain in high-risk children predisposed to perinatal HI. Furthermore, our work aimed to investigate the frequency, location, intensity, and duration of pain in children, analyze sociodemographic factors, and investigate the relationship with child development parameters.

## Materials and methods

Population and study design

An epidemiological, descriptive, cross-sectional study that assesses the prevalence of pain and some of the associated factors in high-risk children predisposed to perinatal HI, including premature babies and children born with a gestational age greater than 35 weeks, was submitted to the Therapeutic Hypothermia Protocol. The study population consisted of newborns discharged from the neonatal intensive care unit of the Pedro Ernesto University Hospital, Rio de Janeiro, Brazil, who were between two and 12 years old and were followed up by a multidisciplinary and transdisciplinary outpatient clinic, according to the model of the project Outpatient Follow-up of High-Risk Newborns (SARAR) of the Faculty of Medical Sciences of the University of the State of Rio de Janeiro. The study was approved by the Research Ethics Committee of the Pedro Ernesto University Hospital in September 2021 (scientific review number: 4,947,839).

Inclusion Criteria

The children included in this study were those followed by the SARAR project after discharge from the NICU, who were between two and 12 incomplete years of age and were born premature with a very low birth weight (less than 1,500 grams) and/or gestational age less than or equal to 32 weeks, or born with a gestational age greater than 35 weeks and diagnosed with perinatal asphyxia and submitted to the therapeutic hypothermia protocol.

In order to participate in the study, it was necessary for the parents to sign the Free and Informed Consent Form and for children aged six years or older to sign the Free and Informed Assent Term.

Exclusion Criteria

Children younger than two years old (the corrected age for prematurity in the case of premature babies) were excluded, considering that the study aims to identify the phenomenon of pain, and the sensory-motor stage, characterized by no mental representation of pain, goes up to approximately two years of age [23].

Data collection and pain assessment

**Figure 1:**
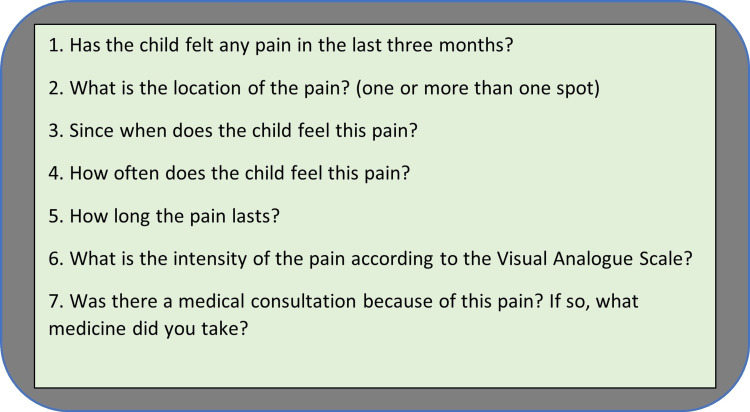
The adapted Lübeck pain-screening questionnaire

Data collection was carried out in one regular outpatient appointment for the assisted child, with the application of a pain assessment questionnaire to the respective guardian as well as filling out a form for collecting sociodemographic, clinical, growth, and development data based on physical and electronic medical records and the Epi Info 7 (CDC, Atlanta, GA) computerized database. The pain assessment instrument was adapted and is based on the Lübeck pain-screening questionnaire, which was specifically designed for an epidemiological study of pain characteristics among children and adolescents [1,2,24]. The questionnaire evaluates if the children have had pain in the last three months and its characteristics, such as frequency, localization (one or more than one spot), duration, intensity, and necessity of medication (Figure 1).

Pain intensity was measured using the visual analog scale (VAS) of pain, which is graduated from zero to 10. Zero represents no pain, and 10 represents the highest pain. Moreover, the consultation of the physical and/or electronic records and computerized database, containing follow-up data, provided anthropometric parameters and developmental assessments, as well as clinical and sociodemographic data.

Statistical analysis

In the statistical analysis, an exploratory analysis of the data was performed with the percentage distribution of categorical and central tendency variables and dispersion for quantitative parameters of a numerical and discrete nature. The percentage distribution of pain was evaluated according to its frequency and intensity. The percentage differences between the evaluated factors and the presence of pain were performed using Fisher's exact test and medians using the non-parametric Wilcoxon rank-sum test, both suitable for small samples. Significance levels of 10% were considered for trends and 5% for statistically significant differences. All statistical analyses were carried out using Microsoft Excel (Microsoft Corporation, Redmond, WA) and R software (R: A language and environment for statistical computing, R Foundation for Statistical Computing, Vienna, Austria).

## Results

Our results show a total of 86 children who agreed to participate in the study: 26 (30%) diagnosed with perinatal asphyxia born with more than 35 weeks gestational hereinafter referred to as the asphyxiation group and 60 (70%) premature. The asphyxiation group is composed of 25 full-term children and one child born at 36 weeks of gestation. The premature group is the one where the babies were born prematurely with a very low birth weight (less than 1,500 grams) and/or gestational age less than or equal to 32 weeks, which are considered high-risk children predisposed to HI. Of the 86 children, 44 were females and 42 were males. As for self-declaration of color, 68 parents and children declared themselves Black and/or Brown, and 18 declared themselves White. Regarding maternal education, 71 mothers had secondary or higher education, 13 had elementary education, and for two, it was not possible to obtain this data (Table 1).

**Table 1:** Description of the participants ¹N=85; ²N=58 (only children with < 6 years); *one or more than one area with a delay DENVER II: children's developmental scale

	Frequency	Percentage (%)
Group		
Asphyxiation	26	30
Premature	60	70
Age (in years)		
< 6 years	60	70
6 years or +	26	30
Sex		
Female	44	51
Male	42	49
Race		
White	18	21
Black/Brown	68	79
Birth Weight		
Appropriate for gestational age	61	70.9
Small for gestational age	3	3.5
Big for gestational age	22	25.6
Birth weight less than 2500 g	60	70
Birth weight bigger than 2500 g	26	30
Central nervous system (CNS)		
CNS imaging without alterations¹	61	72
CNS imaging with alterations¹	24	28
DENVER II		
Normal DENVER II ²	27	47
DENVER II with delay*²	31	53

Regarding the adequacy of birth weight for gestational age, 61 children were classified as adequate for gestational age, 22 were big for gestational age, and three were small for gestational age. Sixty children weighed less than 2,500 grams at birth, while 26 weighed more than 2,500 grams. Furthermore, 64 children were born by cesarean delivery and 22 by normal delivery. As for the alteration of the central nervous system (CNS) measured by an imaging exam (trans-fontanel ultrasound) at birth, 24 children had encephalic alterations. As for the developmental parameters, 31 children showed alterations in the DENVER II test at the follow-up appointment (Table 1).

**Figure 2:**
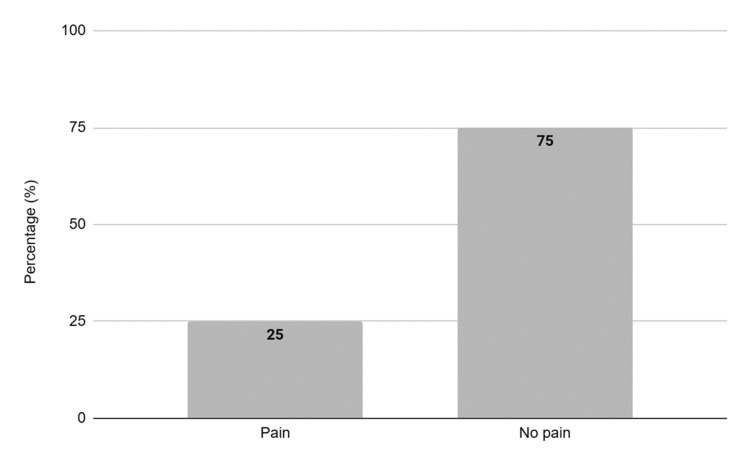
The presence of pain in percentages

The presence of pain was reported in 22 (25%) children (Figure 2), according to the adapted Lübeck Pain-Screening Questionnaire that was applied to their respective guardians.

**Figure 3:**
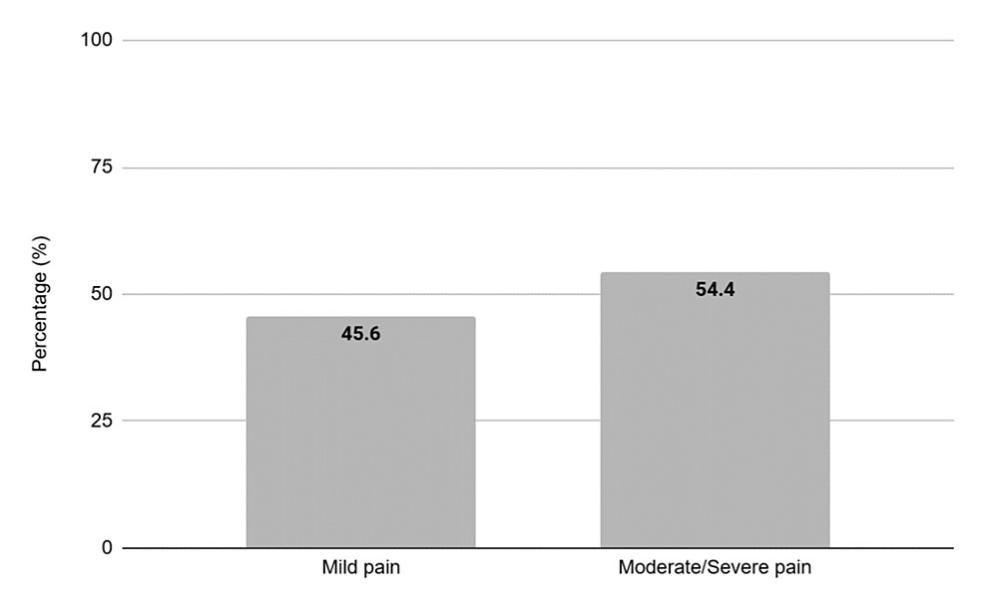
Intensity of pain reported in percentages

Nineteen of whom were premature, and three of the asphyxiation group, 35-week-old or more gestational-age babies, were diagnosed with perinatal HI and submitted to the Therapeutic Hypothermia Protocol. Of the total, 54.4% reported moderate or severe pain (Figure 3).

The head and abdomen were the most reported sites, with a percentage frequency of 36.4%, followed by the lower limbs, upper limbs, and chest (Table 2).

**Table 2:** The pain locations reported by the participants *There were children who reported more than one site of pain

Pain location*	Frequency	Percentage (%)
Head	08	36.4
Abdomen	08	36.4
Lower limbs	07	31.8
Upper limbs	01	4.5
Chest	01	4.5

Of all the children who reported pain, only 11 said they used some medication during this situation for analgesia, with metamizole being the most commonly used medication. The pain was reported more by the female children (68%). Moreover, 10 infants who reported pain were older than six years, and 12 were under six years of age.

The differences were observed in the percentage distribution of pain between the asphyxiation and premature groups (11% vs. 32%; p-value of 0.061 on Fisher's exact test) and in female and male children (34% vs. 17%; p-value of 0.085 on Fisher's exact test) (Table 3).

**Table 3:** Percentage-wise distribution of the presence of pain ¹N=85; ²N=58 (Only children < 6 years); *one or more than one area with a delay DENVER II: children's developmental scale

	With pain		Without pain		p-value
	N	%	N	%	
Group					0.061
Asphyxiation	03	14	23	36	
Premature	19	86	41	64	
Age (in years)					0.282
< 6 years	13	59	47	73	
6 years or +	09	41	17	27	
Sex					0.085
Female	15	68	29	45	
Male	07	32	35	55	
Race					0.544
White	03	14	15	23	
Black/Brown	19	86	49	77	
Central nervous system (CNS)					0.403
CNS imaging without alterations¹	17	81	44	69	
CNS imaging with alterations¹	04	19	20	31	
DENVER II					0.344
Normal DENVER II²	08	62	19	42.2	
DENVER II with delay*²	05	38	26	57.8	

Regarding the pain scale, Black and Brown children had higher median values than White children (p-value<0.027, Wilcoxon rank sum test) (Table 4).

**Table 4:** Measures of central tendency and dispersion of the pain intensity scale (from 0 to 10) ²N=13 (Only children < 6 years); *one or more than one area with a delay DENVER II: children's developmental scale

						p-value
	Average	SD	Median	P25	P75	
Group						0.241
Asphyxiation	3.66	2.08	3.00	2.50	4.50	
Premature	4.97	1.48	4.50	4.00	6.00	
Sex						0.296
Female	5.06	1.70	5.00	4.00	6.00	
Male	4.21	1.22	4.00	4.00	4.75	
Race						0.027
White	3.00	1.00	3.00	2.5	3.50	
Black/Brown	5.09	1.48	5.00	4.0	6.00	
Central nervous system (CNS)						0.157
CNS imaging without alterations¹	5.12	1.56	5.00	4.00	6.00	
CNS imaging with alterations¹	3.62	1.37	3.75	2.75	4.62	
DENVER II						0.888
Normal DENVER II*²	4.18		4.00	3.75	4.87	
DENVER II with delay*²	4.10		4.50	4.00	5.00	

## Discussion

Despite the limited population of our study, it was possible to describe an overview of the pain profile in children with HI, as well as the sociodemographic aspects of the analyzed child population at the Pedro Ernesto University Hospital. In addition, the heterogeneity of the studied group allowed the assessment of pain in children with high risk for both more and less intense hypoxic-ischemic events. Based on the analysis of the results, a higher prevalence of pain was observed among premature infants, females, and Black and Brown children located in the head and abdomen, of moderate and/or severe intensity. Pain is defined as “an unpleasant sensory and emotional experience associated with, or resembling that associated with, actual or potential tissue damage” by the International Association for the Study of Pain [25]. In our study, we considered the intensity of pain mild when ranging from one to three and moderate and/or severe when ranging from four to 10, based on the VAS.

There is a higher prevalence of pain in the premature population, around 32% vs. 11% in the bracket for the two distinct study groups. However, there are no other studies in the literature that have analyzed the population of asphyxiates and premature infants in terms of the pain variable, so this data can be compared. Among the aspects that may be related to this higher prevalence, the immaturity of the descending pain inhibitory pathway as opposed to the full formation of the afferent pathway of pain sensitivity [26] is a risk factor. Furthermore, studies show that premature children, especially those with less than 28 weeks of gestation, tend to stay longer in NICUs and, therefore, are more exposed to painful procedures that cause changes in the brain development process and, consequently, in sensitivity to pain over time [27,28].

As for gender, pain was more prevalent in females, which is already described in the literature in the general population [1], which generates greater restrictions in daily life than in males [24]. In addition, pain was also observed to be more frequently located in the head and abdomen, followed by the lower and upper limbs and chest. These data are similar to what has been observed in other studies, which evaluated the location of pain in children in general, with headaches being the most prevalent [1,24].

Pain intensity is also an interesting aspect to be observed since moderate or severe pain is reported more frequently. These are not unexpected data, as other studies already suggest this prevalence, such as the analysis of pain in children with cerebral palsy [3]. Furthermore, the intensity of pain may also be related to greater impairments in daily life and a greater need to use medication and the health system [24]. In our study, the use of the health system and medication administration due to pain was observed in 50% of the children, a significantly expressive number. It demonstrates the importance of analyzing the location and severity of pain to guide treatment.

It is also worth mentioning that of the 22 children who reported pain, only seven of them reported having this pain for more than three months, configuring it as a condition of chronic pain [25]. This percentage is similar to what is found in the literature in children with so-called major sequelae of HI -cerebral palsy - with around 31% to 43% of the population reporting chronic pain [3, 29]. Regarding the general child population, studies estimate that 25% of children have chronic pain [1], which is therefore below what was observed in our research.

Furthermore, it should be noted that all information was collected according to the guardian's report regarding the characteristics of the children's pain, which ends up expressing a perception of the guardians about the pain, which may not always correspond to the actual characterization of the pain for the children. This limiting aspect was also found in a similar study of the perception of pain in preterm infants with low birth weight, in which a questionnaire was also applied to the children's mothers to obtain data [30]. With this, the discussion arises about the need for further studies with other methodologies for analyzing the pain pattern so that the opinions of these children can be more faithfully reflected.

## Conclusions

All in all, the analysis of pain in children who have a high risk of hypoxic-ischemic events allowed the establishment of an overview of the pain profile in this population. A higher prevalence of pain was observed in premature infants, females, and Black and Brown children, and was located in the head and abdomen, of moderate or severe intensity. Around 32% of children experienced chronic pain due to premature birth or perinatal hypoxia. Therefore, it is notable that pain can be one of the complications resulting from hypoxic-ischemic events, together with retinopathy of prematurity, encephalopathy, and cognitive and/or motor dysfunctions.

The pain, as a possible outcome of this high-risk population, combined with other developmental assessment parameters may contribute to a better understanding of neurological lesions of varying severity in very low birth weight preterm infants and newborns diagnosed with perinatal asphyxia. Future studies can inform the best way to identify and manage pain with effective therapies, such as follow-up with pain clinics, and consequently, an improvement in the quality of life of children with a high risk of HI injury.
